# Efficacy of a Just-in-Time Adaptive Intervention to Promote HIV Risk Reduction Behaviors Among Young Adults Experiencing Homelessness: Pilot Randomized Controlled Trial

**DOI:** 10.2196/26704

**Published:** 2021-07-06

**Authors:** Diane Santa Maria, Nikhil Padhye, Michael Businelle, Yijiong Yang, Jennifer Jones, Alexis Sims, Marguerita Lightfoot

**Affiliations:** 1 Cizik School of Nursing University of Texas Health Science Center at Houston Houston, TX United States; 2 TSET Health Promotion Research Center University of Oklahoma Health Sciences Center Oklahoma City, OK United States; 3 Center for AIDS Prevention Studies and UCSF Prevention Research Center University of California San Francisco San Francisco, CA United States

**Keywords:** youth, homelessness, HIV, prevention, just-in-time adaptive interventions, mHealth, ecological momentary assessments, drug use, stress, intervention, smartphone, mobile phone, drug, efficacy, pilot, feasibility, predictor, risk, behavior

## Abstract

**Background:**

People experiencing homelessness have higher rates of HIV than those who are stably housed. Mental health needs, substance use problems, and issues unique to homelessness such as lack of shelter and transiency need to be considered with regard to HIV prevention. To date, HIV prevention interventions for young adults experiencing homelessness have not specifically addressed modifiable real-time factors such as stress, sexual or drug use urge, or substance use, or been delivered at the time of heightened risk. Real-time, personalized HIV prevention messages may reduce HIV risk behaviors.

**Objective:**

This pilot study tested the initial efficacy of an innovative, smartphone-based, just-in-time adaptive intervention that assessed predictors of HIV risk behaviors in real time and automatically provided behavioral feedback and goal attainment information.

**Methods:**

A randomized attention control design was used among young adults experiencing homelessness, aged 18-25 years, recruited from shelters and drop-in centers in May 2019. Participants were randomized to either a control or an intervention group. The intervention (called MY-RID [Motivating Youth to Reduce Infection and Disconnection]) consisted of brief messages delivered via smartphone over 6 weeks in response to preidentified predictors that were assessed using ecological momentary assessments. Bayesian hierarchical regression models were used to assess intervention effects on sexual activity, drug use, alcohol use, and their corresponding urges.

**Results:**

Participants (N=97) were predominantly youth (mean age 21.2, SD 2.1 years) who identified as heterosexual (n=51, 52%), male (n=56, 57%), and African American (n=56, 57%). Reports of sexual activity, drug use, alcohol use, stress, and all urges (ie, sexual, drug, alcohol) reduced over time in both groups. Daily drug use reduced by a factor of 13.8 times over 6 weeks in the intervention group relative to the control group (Multimedia Appendix 4). Lower urges for sex were found in the intervention group relative to the control group over the duration of the study. Finally, there was a statistically significant reduction in reports of feeling stressed the day before between the intervention and control conditions (*P*=.03).

**Conclusions:**

Findings indicate promising intervention effects on drug use, stress, and urges for sex in a hard-to-reach, high-risk population. The MY-RID intervention should be further tested in a larger randomized controlled trial to further investigate its efficacy and impact on sexual risk behaviors.

**Trial Registration:**

ClinicalTrials.gov NCT03911024; https://clinicaltrials.gov/ct2/show/NCT03911024

## Introduction

### Background

Young adult homelessness continues to be a major public health problem with 1 in 10 young adults aged 18 to 25 years experiencing homelessness over the course of a year [1] and an estimated 1.7 to 2.5 million youth under 25 years experiencing homelessness each year in the United States [2]. Securing food and shelter while experiencing the hardships and dangers of living on the streets creates enormous challenges to maintaining one’s health and well-being [3]. The mortality rate for youth experiencing homelessness (YEH) is 5 to 10 times higher than peers in the general population [4], and many YEH have chronic mental and physical conditions, engage in substance use, and have unmet health and mental health care needs [3,5,6]. One persistent health concern is HIV. Unstable housing is a significant barrier to accessing and engaging in HIV care, maintaining viral suppression, and reducing HIV transmission [7]. The implications of mental health needs, substance use problems, and issues unique to YEH such as the lack of stable sheltering options need to be considered with regard to HIV prevention. This is particularly salient as people experiencing homelessness have higher rates of HIV than those who are stably housed [8]. While HIV prevalence data for YEH are sparse, one study found a self-reported HIV diagnoses rate of 4% [9].

### HIV Risk Among Youth

HIV risk among all youth is correlated with sexual orientation [10], childhood abuse [11,12], and histories of foster/juvenile justice involvement [13-16]. Further, modifiable factors have been found to predict HIV risk in nonhomeless youth populations. Stress, sexual urge, and substance use negatively impact sexual decision-making and increase HIV risk [17-19]. HIV risk behaviors such as condomless sex and substance use are also correlated with factors such as stress [20,21] and depression [22]. Stress has also been correlated with inconsistent condom use; number of sexual partners; and substance use in young females [23-27], African American adolescent females [28], urban Black heterosexual men [29], and young men who have sex with men (MSM) [30-32]. Finally, a history of traumatic stress and current substance use are associated with more frequent sexual urges among MSM [33].

Young adults engage in HIV risk behaviors [17-19]. Engagement in risk behaviors may be heightened by low motivation for HIV prevention related to time spent on the streets [18] and exacerbated by high levels of trauma experienced prior to and while homeless [34,35]. In a systematic review of sexual behaviors among YEH, most studies did not examine how situational variables affect sexual risk [34] despite the mounting evidence of the significant correlations between elevated stress, sexual urge, and substance use with HIV risk behaviors in female youth [26,27,36,37] and YEH [38,39]. For example, experiencing sexual urges has been found to influence YEH’s decision to engage in condomless sex [40]. Substance use is also associated with condomless sex and sexual victimization among homeless and urban youth [35,41,42]. Finally, in one study using ecological momentary assessments (EMAs), the odds of having sex on a given day were found to be highest on days when YEH experienced sexual urge and used drugs, and the odds of substance use were highest on the days when youth experienced high stress and drug urge [43].

### HIV Prevention Among YEH

To date, HIV prevention interventions for YEH have not specifically addressed modifiable real-time factors such as stress, sexual urge, or substance use, or been delivered at the time of heightened risk. Using real-time, personalized HIV prevention messages may provide more timely information and produce more motivation for behavioral change than those seen in prior interventions. Importantly, when programs are tailored and relevant, YEH are interested in health promotion programs, can be recruited and retained in interventions and research studies [44,45], and demonstrate improved outcomes despite challenges with sustaining intervention engagement. In a group-based study with YEH, there were positive intervention effects on alcohol use, motivation, and condom self-efficacy even though 52% of participants did not attend all intervention sessions [46].

One method to reach and engage YEH in interventions is to utilize mobile technology. Interventions that provide personal motivational messages in response to real-time thoughts, feelings, sexual urges, and substance use may be more effective than interventions that are homogeneous and primarily informational in nature [47,48]. Interventions that can be delivered via smartphones at the time of heightened HIV risk may place tailored health messages more proximally to critical behavioral decision points, thereby increasing access to and relevance of prevention interventions [49].

### Assessing Real-Time HIV Risk

EMA is currently the gold standard and most accurate way to measure real-time factors in natural settings [50,51], with high compliance rates (78%) found among youth across 42 studies [52]. EMA assesses within-person variance to risk exposures (eg, where, when, and with whom sexual risk is likely to occur throughout a day) by capturing repeated measures to assess changes in behaviors, cognition, environmental factors, and symptoms. Therefore, EMA has the potential for use as a driver of interventions that tailors messages to one’s current risk level. Affect and behavioral monitoring associated with EMA may also increase self-awareness of HIV risk by capturing behavioral patterns and assessing predictors of risk, including sexual urge, stress, and substance use [53]. In smoking studies, EMA alone has lowered urges and stress [54]. By increasing one’s awareness of risk, EMA can detect risk before the behavior occurs. EMA data that are collected at or near the moment when HIV risk behaviors occur can reduce recall biases that are associated with other measures. For example, one study found that 54% of youth reported condom use during their last sexual encounter at baseline, yet 76% of sex acts were condomless when assessed in real time using EMA [46]. Recall data have higher potential for bias, neglect intraindividual variability, and do not capture risk and protective factors as they occur in the real world. Consistently high EMA completion rates have also been found among youth, including substance use (80%) [55], substance use recovery (87%) [56], smoking (88%) [57], sexual behaviors (80%) [58], and drinking (89%) [59]. Little is known about EMA compliance rates among YEH, although one study found that 89% of participants provided EMA data during the study with a 62% compliance rate [60].

### Delivering Interventions in Response to Real-Time Risk

Just-in-time adaptive interventions (JITAIs), such as EMA-driven messages, may be an effective delivery strategy for information and motivational messages to be delivered at the time of heightened risk detection prior to engaging in a risk behavior and in response to a reported risk behavior antecedent. JITAIs can target the proximal, modifiable mediators that indicate the emergence of a vulnerable state (eg, high sexual urge, substance use, or spikes in stress). For example, messages can be delivered in response to elevated sexual urge and/or after a recent sexual assault [43,60-62]. JITAIs can be effectively delivered for a variety of health behaviors and psychological symptoms management [63], and real-time messaging has improved risk behaviors, including long-term smoking cessation [64], binge drinking among young adults with hazardous alcohol use [65], and sexual risk behaviors and sexually transmitted infection (STI) testing among youth [66]. JITAIs have been found to significantly reduce anxiety and stress [67-69], alcohol use [70], and depressive symptoms [71], and increase pre-exposure prophylaxis (PrEP) uptake among MSM [72]. EMA-informed JITAIs build off of the willingness to disclose personal information electronically through EMAs [73], overcome geographic and organizational barriers to reaching the underserved [74], require few agency resources, are easily accessible to youth, address personalized prevention care, and are particularly attractive to young people especially when these interventions are developed with the target audience to enhance sustainable use [75].

Optimizing the interactivity that smartphones provide, it is possible to combine tested mobile health (mHealth) strategies (eg, text messaging) [76] with other proven technology-based strategies such as tailored education [77,78] and motivational messaging [79,80] to deliver scalable, cost-efficient HIV prevention interventions. These strategies have had positive outcomes for smoking cessation in youth [81] and among homeless adults [82,83], treatment adherence among youth living with HIV [78], and HIV prevention in African American youth [84,85]. Such real-time interventions may address the challenges of reaching YEH related to transiency and heterogeneity [86] by targeting real-time factors such as sexual urge, substance use, and stress at the time of heightened HIV risk [34]. JITAIs can deliver personalized HIV prevention messages that vary in content and dose depending on an individual’s current sexual urges, substance use, and spikes in stress [87], providing the right type and dose at the optimal time [88]. To our knowledge, no JITAIs have been developed for YEH that use EMA and deliver personalized, time-varied HIV prevention messages. This study advances work done in this field by evaluating the preliminary efficacy of an EMA-driven, personalized HIV prevention intervention that is sensitive to variability in risk among a sample of YEH.

### Purpose of This Study

No interventions to date have been carried out to intervene at the individual level to increase HIV risk perception and behavioral self-monitoring for the prevention of drug use and sexual risk behaviors among YEH. Therefore, in this study we leveraged the use of mobile technology to test the initial efficacy of a beta-tested mobile, just-in-time, adaptive HIV prevention messaging intervention called MY-RID (Motivating Youth to Reduce Infection and Disconnection; pronounced “My Ride”) among YEH on sexual behaviors and substance use. MY-RID is an innovative, theory-based (Information, Motivation, and Behavioral Skills [IMB] model [89]) JITAI, delivered via a smartphone app, that targets real-time predictors of HIV risk behaviors at the time of high risk, responds to patterns of risk behaviors to motivate participants to engage in prevention services, and provides behavioral feedback and goal attainment information in real time [88].

## Methods

### Study Design

The study used a 1:1 randomized attention control design to pilot test the initial efficacy of MY-RID. All study protocols were approved by the Institutional Review Board for the Protection of Human Subjects prior to recruitment. Interested youth were screened, consented, and then completed a baseline survey (Multimedia Appendix 1) about demographic characteristics that was administered on an iPad. Participants were then randomized to either the control or the intervention group, and every participant received an Android smartphone with unlimited data. The phone was preloaded with the MY-RID app, which uses the INSIGHT mHealth platform [90]. The MY-RID app prompted and delivered once daily EMAs and targeted messages (ie, intervention messages or control condition messages) in response to EMA data for 6 weeks. All participants were oriented to the EMA procedures and indicated the time they typically woke up and went to bed each day to assure EMA and messages did not disturb their sleep.

### Recruitment

YEH aged 18-25 years were recruited from a shelter and drop-in center serving YEH over the course of 1 week in May 2019 from the Houston, TX, region using group presentations and flyers. Youth were eligible if they were between 18 and 25 years and were experiencing homelessness. Experiencing homelessness was defined as: (1) living on the streets or in a place not meant for human habitation, a shelter, hotel/motel, or any place they cannot stay for more than 30 days or (2) currently receiving homelessness services. In order to assure participants could complete the EMA surveys and understand the tailored intervention messages independently, participants were required to be able to speak and read English (determined by the Rapid Estimate of Adult Literacy in Medicine-Short Form [REALM]) at a 7th-grade level or higher [91].

### Ecological Momentary Assessments

Participants were prompted 3 times a day to complete EMAs during the first 2 weeks of the study, two daily EMAs for the next 2 weeks, and one daily EMA for the final 2 weeks. These EMAs took about 1 to 2 minutes to complete. Example EMA items included: “I am feeling a strong urge to: (have sex, do drugs, drink alcohol),” “Select all of the drugs you used in the last 2 hours,” “Did you have sex yesterday?” EMAs were prompted on the phone 30 minutes after the participants indicated wake time and took approximately 1 to 5 minutes to complete. All participants were able to receive up to $120 based on their EMA response rate (>90%=$40, 75%-89%=$35, 50%-74%=$30, 25%-49%=$20, and >24%=$15) over the 6-week period. A compensation monitor was programmed in the app to show the participants their response and compensation.

### Intervention Messages

Using the IMB model to assure the messaging reflected on the cognitive, behavioral, and environmental factors of HIV [89], over 360 messages were developed and beta tested with YEH that addressed the real-time predictors of HIV risk, including urge to use drugs, sexual urge, stress, and drug use. First, the research team developed the theory-driven messages. Next, messages were presented to YEH, who offered ways they would “say to this to a friend” to increase the linguistic relevance of the messages. Finally, messages were beta tested with YEH until there was agreement that the message was likely to impact thoughts and behaviors. Example messages included: “Avoiding drugs can help reduce mental health issues.” and “It’s easy to catch an STD. Be careful and always use a condom.” Consistent with the IMB model, messages included information and knowledge about the behavior in question (eg, substance use, sexual risk behavior), the individual’s motivation to perform the behavior, and the behavioral skills necessary to perform the behavior [92].

### Intervention Arm

During the baseline visit, youth randomized to the intervention arm were asked to set an HIV prevention behavior goal after a study team member reviewed basic HIV prevention strategies. Goal options included increasing condom use, decreasing the number of sexual partners, using PrEP daily, getting tested for HIV, reducing drug and alcohol use, not having sex while using drugs or drinking alcohol, and avoiding injection drug use. Data entered by the participant during the daily EMAs populated a graphic goal interface that depicted the current level of goal attainment based on EMA data. This interface was accessible on the phone at any time by the participant. After the completion of each EMA, participants in the intervention group received tailored messages that addressed the participant’s goals: (1) safer sexual behaviors, (2) alcohol or drug use, (3) PrEP interest, and (4) HIV testing. Messages were selected using an algorithm that prompted messages based on current risk factors that targeted reduction in alcohol and drug use, promoted condom use, and provided urge management strategies (Multimedia Appendix 2). Generalized linear mixed models revealed several predictors of engagement in sexual activity that could increase HIV risk, including constant characteristics (race, sexual orientation), diagnosed conditions (psychosis, posttraumatic stress disorder), and time-varying predictors (urge for sex, drug use) [43]. Predictors of drug use were all time varying: urge for drug use, urge for alcohol use, urge to steal, viewing pornography, alcohol use, and experience of discrimination [60].

### Attention Control Arm

Participants in the attention control condition were asked to set a behavioral goal related to general health behaviors. Goal options included getting 7 or more hours of sleep, eating 5 or more servings of fruits or vegetables, not using tobacco products, and exercising for at least 60 minutes a day. The control group answered the same EMA items as the intervention group; however, after the completion of each EMA, the control group received different messages related to healthy nutrition, physical activity, sleep hygiene, and prevention of tobacco use. These messages were not tailored to the EMA but sent in a random order.

### Measures

The baseline and 6-week follow-up survey assessed the demographics that have been associated with sexual risk among YEH such as gender identity, age, education, sexual orientation, and adverse childhood experiences [93,94]. Baseline measures also assessed HIV/STI testing, stress [95], depression [96], and psychological distress [97], all of which have been validated previously in YEH studies. EMA measures assessed real-time HIV risk behaviors, including stress, sexual behaviors, sexual urge, and substance use. The EMA items utilized Likert scales and were developed from prior EMA studies [50,98] and tested in studies within the YEH population [43,60].

### Statistical Analyses

Participant retention was evaluated to inform future JITAI studies with YEH. Kaplan-Meier survival functions were calculated using the survival package [99] in the R programming language (R Core Team) [100] to obtain the median participation time in the control and intervention groups, based on the total time spent completing activities in the app. Participant EMA completion rates were also examined over the 6-week study duration.

Baseline data consisting of participants’ demographics and health status measures were compared between the control and intervention groups using Pearson chi-square tests. In instances of low cell counts, the Fisher exact test was calculated using SPSS Statistics 26 (IBM Corp). Outcomes were analyzed first on the basis of cumulative counts before using longitudinal models. The counts provided the number of participants who engaged in sexual activity, or substance use, or reported urges, or reported being stressed at least once over the 6-week study period. The differences in proportions between the control and intervention groups were assessed with Pearson chi-square tests or the Fisher exact test. 

Longitudinal analysis was carried out with Bayesian hierarchical logistic regression models that assessed the intervention effects on engaging in sex, drug use, alcohol use, and their corresponding urges. The intervention effect was modeled with fixed effects that included the interaction of intervention group and time. Random intercept and slope allowed the intervention effect to vary among participants. Stress experienced at the time of response and stress experienced by participants on the previous day were analyzed in a similar manner. Information about the prior distributions and more details about the analysis are provided in Multimedia Appendix 3. The solution was implemented with the *RStan* package [101] via code written and executed in the RStudio environment (RStudio, PBC) [102].

Sensitivity analysis was conducted to check the robustness of statistically significant intervention effects that resulted from the hierarchical regression models. The models were expected to provide robust intervention effects if data were missing at random. We considered the possibility of nonignorable missing mechanisms and used a tipping-point approach for the sensitivity analysis [103]. Multimedia Appendix 3 presents more information about the missingness mechanisms and sensitivity analysis.

## Results

A total of 100 participants were enrolled, of which 3 were excluded due to lack of EMA data. Data were analyzed for the remaining 97 participants aged 18-25 years (mean 21.2, SD 2.1 years), of whom 48 (49.5%) were randomly assigned to the intervention group and 49 (50.5%) were randomly assigned to the control group. Over half of participants identified as heterosexual (n=51, 52%), male (n=56, 57%), and African American (n=56, 57%). There was noteworthy diversity in gender identity, sexual orientation, and racial identity (Table 1). Additionally, 40% (n=39) of participants had been involved with the juvenile justice system. Over 35% (n=37) of the participants rated their overall health status as excellent or very good, and 52% (n=51) of participants had been tested for HIV within the past 3 months. There were no statistically significant differences between the groups in terms of race or ethnicity, gender identity, HIV risk behaviors, or any of the other variables that were collected at baseline.

Kaplan-Meier survival curves showed that participants engaged with the app for 34.5 days (median), or about 5 of 6 possible weeks (95% CI 28.5-39.5). The length of participation was based on the range from the first to last time of responses received from each participant, and the median value was identical in the control and intervention groups. Another metric of participation was the participant response rate, which decreased over time, resulting in a median of 19 days (IQR 25) of useful data per participant. That corresponds to 46.3% of the maximum potential for responses, or 56.7% of the response potential during the median participation time. The median times for responding to EMAs were 160 seconds (IQR 98) for the daily EMA and 87 seconds (IQR 46) for the random assessments.

Frequencies of participant reported behaviors, urges (ie, sex, drug, alcohol), and stress during the EMA daily diaries (Table 2) revealed that there was a statistically significant (*P*=.03) difference in reports of feeling stressed the day before with a lower proportion of participants in the intervention group reporting feeling stressed the day before compared with control group participants (n=20, 41.7% vs n=31, 63.3%) across the 6 weeks of EMA implementation. Although not statistically significant, the intervention group displayed fewer transactional sex behaviors relative to the control group (n=5, 10.4% vs n=9, 18.4%). HIV risk behaviors were similar across gender identities, but current stress was higher among transgender/genderqueer participants compared to cisgender males and females (Table 3).

**Table 1:** Baseline characteristics by group.

Variable		EMA^a^ participants (N=97)			*P* value^b^	
		Intervention group (n=48), n (%)	Control group (n=49), n (%)			
**Age**					.62	
	18-21 years	28 (58.3)	31 (63.3)			
	22-25 years	20 (41.7)	18 (36.7)			
**Gender**					.39	
	Male	25 (52.1)	31 (63.3)			
	Female	20 (41.7)	14 (28.6)			
Transgender/genderqueer/other/missing		3 (6.3)	4 (8.2)			
**Sexual orientation**					.12	
	Gay	1 (2.1)	7 (14.3)			
	Lesbian	4 (8.3)	2 (4.1)			
	Straight (ie, not gay)	29 (60.4)	22 (44.9)			
	Bisexual	9 (18.8)	14 (28.6)			
	Asexual/pansexual/other	5 (10.4)	4 (8.2)			
**Race or ethnicity**					.06	
	White or Caucasian	0 (0)	3 (6.1)			
	Black or African American	31 (64.6)	25 (51.0)			
	Hispanic or Latino	7 (14.6)	2 (4.1)			
	American Indian/Asian or Pacific Islander/other	5 (10.4)	9 (18.4)			
	Multiracial	5 (10.4)	10 (20.4)			
Involved with the juvenile justice system		19 (39.6)	20 (40.8)		.90	
**Age of first homelessness**						.07
	Minor (<18 years)	27 (56.3)	18 (36.7)			
	Adult (≥18 years)	21 (43.8)	30 (61.2)			
	Missing	0 (0)	1 (2.0)			
**Mental health**					.68	
	Excellent	13 (27.1)	13 (26.5)			
	Very good	6 (12.5)	5 (10.2)			
	Good	10 (20.8)	16 (32.7)			
	Fair	13 (27.1)	9 (18.4)			
	Poor	6 (12.5)	5 (10.2)			
	Missing	0 (0)	1 (2.0)			
**Emotional problems in the last 7 days**						.30
	Never	4 (8.3)	2 (4.1)			
	Rarely	7 (14.6)	7 (14.3)			
	Sometimes	19 (39.6)	16 (32.7)			
	Often	7 (14.6)	16 (32.7)			
	Always	11 (22.9)	8 (16.3)			
**Last HIV test**					.88	
	Within the past 3 months	24 (50.0)	27 (55.1)			
	More than 3 months	16 (33.3)	15 (30.6)			
	Never been tested for HIV	8 (16.7)	7 (14.3)			

^a^EMA: ecological momentary assessment.

^b^Calculated using the Pearson chi-square test or the Fisher exact test for differences of proportion between the intervention and control groups.

**Table 2:** Frequency of participants who reported specific HIV risk factors by treatment group.

Variable	EMA^a^ participants^b^		*P* value^c^
	Intervention group, n (%)	Control group, n (%)	
Had sex yesterday	33 (68.8)	29 (59.2)	.33
Used drugs yesterday	25 (52.1)	24 (49.0)	.76
Sex behaviors with drug use	18 (37.5)	15 (30.6)	.47
Condomless sex	8 (16.7)	14 (28.6)	.16
Urge to have sex	36 (75.0)	34 (69.4)	.54
Urge to use drug	25 (52.1)	30 (61.2)	.36
Traded sex yesterday	5 (10.4)	9 (18.4)	.27
Felt stressed yesterday	20 (41.7)	31 (63.3)	.03
Feel stressed now	30 (62.5)	36 (73.5)	.25

^a^EMA: ecological momentary assessment.

^b^Number of participants who engaged in the behavior at least once.

^c^Chi-square test was fit to test the significance of differences between the intervention and control groups. Significance level at *P*<.05.

**Table 3:** Frequency of participants who reported specific HIV risk factors by gender identity.

Variable	EMA^a^ participants^b^				*P* value^c^
	Cis-male (n=56), n (%)	Cis-female (n=34), n (%)	Transgender, gender queer, or other (n=7), n (%)		
Had sex yesterday	36 (64.3)	22 (64.7)	4 (57.1)	.93	
Used drugs yesterday	27 (48.2)	18 (52.9)	4 (57.1)	.85	
Sex with drug use	20 (35.7)	10 (29.4)	3 (42.9)	.73	
Condomless sex	12 (21.4)	9 (26.5)	1 (14.3)	.74	
Urge to have sex	39 (69.6)	26 (76.5)	5 (71.4)	.78	
Urge to use drug	28 (50.0)	21 (61.8)	6 (85.7)	.15	
Traded sex yesterday	9 (16.1)	4 (11.8)	1 (14.3)	.85	
Felt stressed yesterday	24 (42.9)	22 (64.7)	5 (71.4)	.08	
Feel stressed now	32 (57.1)	28 (82.4)	6 (85.7)	.03	

^a^EMA: ecological momentary assessment.

^b^Number of participants who engaged in the behavior at least once.

^c^Chi-square test was fit to test the significance of differences between the intervention and control groups. Significance level at *P*<.05.

**Figure 1:**
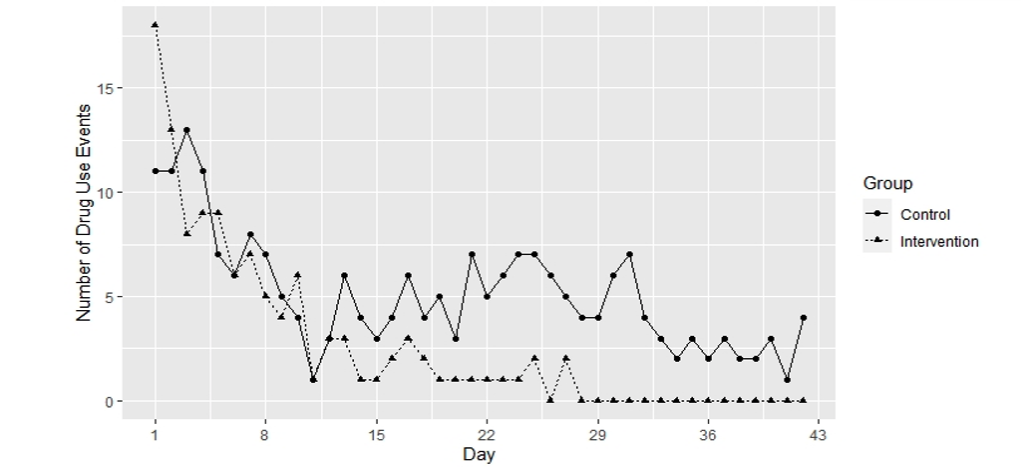
Daily totals of participants who reported using drugs over the 6-week study period, shown separately for the control and intervention groups of the study.

Having sex, drug use, and alcohol use reduced over the 6-week EMA period in both groups (Figure 1 and Table 4). An intervention effect was observed for drug use. The odds ratio (OR) for the interaction of the intervention and time measured on the log scale (OR 0.62, 95% CI 0.39-0.97) implied that the odds of drug use reduced by a factor of 13.8 over 6 weeks, or by a factor of 8.5 over 3 weeks (mid-way point), in the intervention group relative to the control group. There was no intervention effect for having sex or alcohol use.

**Table 4:** Parameter estimates of fixed and random effects arising from Bayesian hierarchical logistic regression models for sexual intercourse, drug use, and alcohol use.

Variable			Coefficient, mean (SD)		Odds ratio (95% CI)		ESS^a^		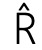 ^b^
**Outcome: sexual intercourse**									
	Intercept	–0.863 (0.41)		0.422 (0.181-0.923)		1000		1.00	
	Days (log)	–0.601 (0.127)		0.548 (0.420-0.687)		563		1.00	
	Intervention	–0.098 (0.554)		0.907 (0.299-2.740)		1366		1.00	
	Intervention × days (log)	0.04 (0.158)		1.041 (0.769-1.423)		1321		1.00	
	Random intercept: σ	1.758 (0.285)		—^c^		1708		1.00	
	Random slope: σ	0.361 (0.098)		—		438		1.00	
**Outcome: drug use**									
	Intercept	–1.669 (0.663)		0.188 (0.047-0.614)		872		1.00	
	Days (log)	–0.597 (0.17)		0.550 (0.379-0.743)		737		1.00	
	Intervention	0.779 (0.861)		2.179 (0.415-12.231)		860		1.00	
	Intervention × days (log)	–0.486 (0.233)		0.615 (0.386-0.971)		1548		1.00	
	Random intercept: σ	3.212 (0.502)		—		1180		1.00	
	Random slope: σ	0.519 (0.138)		—		478		1.00	
**Outcome: alcohol use**									
	Intercept	–2.185 (0.541)		0.112 (0.037-0.303)		673		1.01	
	Days (log)	–0.564 (0.181)		0.569 (0.392-0.795)		672		1.01	
	Intervention	0.115 (0.652)		1.122 (0.312-4.035)		1533		1.00	
	Intervention × days (log)	–0.139 (0.23)		0.870 (0.545-1.344)		1603		1.00	
	Random intercept: σ	1.858 (0.418)		—		651		1.00	
	Random slope: σ	0.542 (0.14)		—		557		1.01	

^a^ESS: effective sample size; after accounting for autocorrelated samples.

^b^Potential scale reduction statistic; 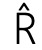
<1.1 indicates convergence of Markov chains.

^c^Not applicable.

Urges for sex, drugs, or alcohol reduced over the study period in both groups (Table 5). Lower odds of the urge for sex were found in the intervention group (OR 0.16, 95% CI 0.04-0.63), and this was a time-independent effect that indicated suppressed sexual urge in the intervention group over the duration of the study. There was no intervention effect for drug or alcohol urge. Stress experienced now and the day before was also reduced over time in both groups (Table 6), with no intervention effect.

**Table 5:** Parameter estimates of fixed and random effects arising from Bayesian hierarchical logistic regression models for urges: urge for sex, urge for drug use, and urge for alcohol.

Variable			Coefficient, mean (SD)		Odds ratio (95% CI)		ESS^a^		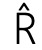 ^b^
**Outcome: urge for sex**									
	Intercept	1.115 (0.495)		3.050 (1.157-8.004)		1757		1.00	
	Days (log)	–0.807 (0.145)		0.446 (0.333-0.586)		1490		1.00	
	Intervention	–1.845 (0.709)		0.158 (0.038-0.626)		2367		1.00	
	Intervention × days (log)	0.352 (0.206)		1.422 (0.947-2.143)		2141		1.00	
	Random intercept: σ	2.157 (0.358)		—^c^		2229		1.00	
	Random slope: σ	0.535 (0.105)		—		1379		1.00	
**Outcome: urge for drugs**									
	Intercept	–0.846 (0.582)		0.429 (0.130-1.296)		1427		1.00	
	Days (log)	–0.537 (0.171)		0.584 (0.410-0.799)		1512		1.00	
	Intervention	0.291 (0.809)		1.338 (0.274-6.430)		1920		1.00	
	Intervention × days (log)	–0.354 (0.250)		0.702 (0.428-1.148)		2499		1.00	
	Random intercept: σ	2.469 (0.445)		—		1283		1.00	
	Random slope: σ	0.611 (0.137)		—		1065		1.00	
**Outcome: urge for alcohol**									
	Intercept	–1.318 (0.709)		0.268 (0.057-0.944)		1475		1.00	
	Days (log)	–0.767 (0.207)		0.464 (0.297-0.679)		1272		1.00	
	Intervention	0.634 (0.930)		1.885 (0.313-12.884)		2500		1.00	
	Intervention × days (log)	–0.305 (0.294)		0.737 (0.406-1.279)		2892		1.00	
	Random intercept: σ	2.663 (0.544)		—		1494		1.00	
	Random slope: σ	0.616 (0.171)		—		772		1.01	

^a^ESS: effective sample size; after accounting for autocorrelated samples.

^b^Potential scale reduction statistic; 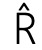
<1.1 indicates convergence of Markov chains.

^c^Not applicable.

**Table 6:** Parameter estimates of fixed and random effects arising from Bayesian hierarchical logistic regression models for stress experienced now and stress experienced the day before.

Variables			Coefficient, mean (SD)		Odds ratio (95% CI)		ESS^a^		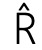 ^b^
**Outcome: stressed now**									
	Intercept	1.861 (0.674)		6.430 (1.779-25.229)		2031		1.00	
	Days (log)	–0.762 (0.199)		0.467 (0.310-0.681)		1904		1.00	
	Intervention	1.448 (1.015)		4.255 (0.584-32.169)		2044		1.00	
	Intervention × days (log)	–0.107 (0.304)		0.899 (0.495-1.642)		2191		1.00	
	Random intercept: σ	3.373 (0.570)		—^c^		1623		1.00	
	Random slope: σ	0.902 (0.158)		—		1766		1.00	
**Outcome: stressed yesterday**									
	Intercept	–0.216 (0.418)		0.806 (0.347-1.831)		1591		1.00	
	Days (log)	–0.339 (0.149)		0.712 (0.526-0.942)		1445		1.00	
	Intervention	–0.027 (0.597)		0.973 (0.300-3.238)		2030		1.00	
	Intervention × days (log)	0.012 (0.213)		1.012 (0.670-1.540)		1984		1.00	
	Random intercept: σ	1.826 (0.417)		—		899		1.01	
	Random slope: σ	0.623 (0.129)		—		1050		1.01	

^a^ESS: effective sample size; after accounting for autocorrelated samples.

^b^Potential scale reduction statistic; 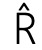
<1.1 indicates convergence of Markov chains.

^c^Not applicable.

The number of participants with any PrEP use during the study was 17%—9 (18%) in the control group and 8 (16%) in the intervention group (χ^2^_1_=0.07, *P*=.79). HIV tests were completed by 11 (22%) and 13 (26%) participants in the control and intervention groups, respectively (χ^2^_1_=0.22, *P*=.64). Intervention goals were selected by participants at the baseline visit. Overall, participants chose from goals, including increasing condom use (n=13), reducing drug and alcohol use (n=9), reducing the number of sex partners (n=9), increasing HIV testing (n=7), using PrEP for HIV prevention (n=6), reducing injection drug use (n=4), and reducing sex while under the influence of drugs or alcohol (n=2).

**Figure 2:**
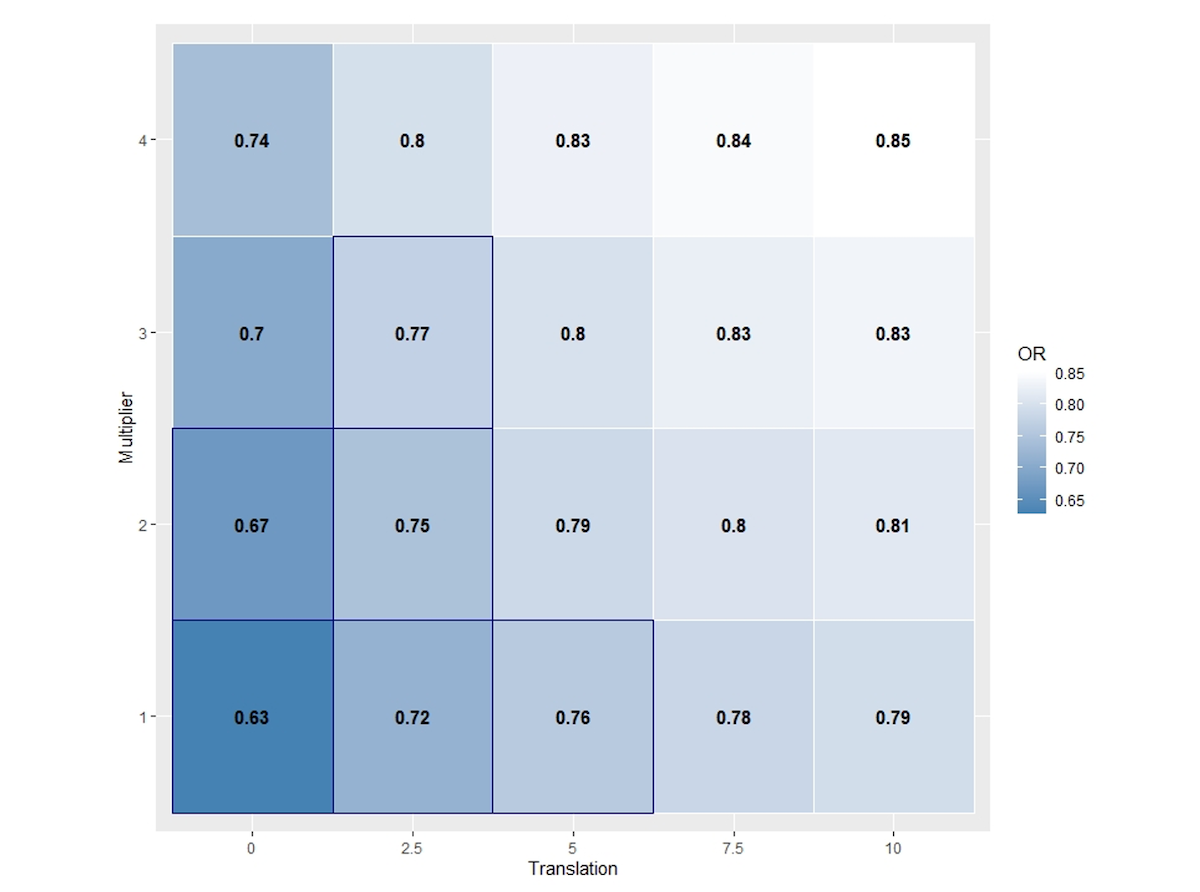
Tipping-point display of the sensitivity analysis for drug use that is based on two nonignorable missingness mechanisms. Each 2.5-unit increment in the translation levels represents roughly one unreported drug use event per nonuser during the study period. The multipliers are inflation factors of the probability of drug use on nonresponse days among those with a record of drug usage. The numbers in each cell display the odds ratio (OR) of the intervention effect, that is, the interaction of group and time. The cells with a thick navy border have *P*<.05. The OR and *P* values represent a summary of results from the multiple imputation.

Sensitivity analysis with the tipping-point approach showed that the intervention effect for drug use was fairly robust on both dimensions of the nonignorable missing mechanisms (Figure 2). The intervention effect was significant even if there was an average of two unreported drug use events among participants who never reported drug use. Similarly, the intervention effect could withstand a doubling of the probability of drug use on nonresponse days among drug users. The intervention effect could also withstand a combination of one unreported drug use event among nonusers and doubling of the probability on the other dimension. The intervention effect tipped over into nonsignificance (at the 95% confidence level) if there were two unreported drug use events simultaneously with a doubling of the probability of drug use in the second mechanism.

## Discussion

### Principal Findings

This pilot randomized controlled trial of a theoretically based intervention adds to the growing body of literature on the feasibility and potential efficacy of JITAIs to address the modifiable risk factors that are correlated with HIV risk. The findings suggest promising preliminary effects of MY-RID among YEH compared with an attention control group. This intervention was effective at reducing (1) the odds of substance use, (2) the urge for sex, and (3) stress among YEH. This is the first evidence that JITAIs can reduce drug use among a high substance-using population of YEH. Given the association between substance use, as well as sexual risk behaviors (eg, condomless sex) and sexual victimization, among homeless and urban youth [35,38,39,41,42], MY-RID may be a promising approach to HIV prevention in a hard-to-reach population. These findings add to the mounting evidence of the malleability of the risk factors that impact HIV risk in real time, including stress, sexual urge, and substance use [26,27,36-39].

While this study was not powered to detect small effects or examine the impact of the intervention on condom use, the data suggest that experiencing sexual urges can negatively influence the use of condoms [40]. In addition, only 13 (27%) participants chose increasing condom use as their behavioral goal, which may also account for the lack of impact of the intervention on condomless sex. As well, while we did not examine the intervention effect on engaging in sex while using substances, the substance use literature reports that sex is highest on substance-using days [43] and that substance use is also associated with sexual risk behaviors (eg, condomless sex) and sexual victimization among youth [35,41,42].

Findings from this study further suggest that MY-RID may reduce mental health symptoms such as stress and urge, which are antecedents to HIV risks. JITAIs have been found to improve psychological symptoms [63], anxiety and stress [67-69], and depressive symptoms [71]. The evidence linking stress to sexual risk behaviors is growing [104]. Delivering coping messages when symptoms (eg, drug or alcohol use urges) are reported may improve stress management and reduce HIV risk behaviors such as substance use and risky sexual activity. Intervention effects were also found for reducing urges for sex among YEH. While reductions in sexual activity and alcohol did not reach significance, they decreased throughout the intervention period in both groups. This may indicate the need to conduct an adequately powered randomized trial to determine if these reductions in risk are significant and sustained over time.

While EMA completion rates were low among this sample of YEH (ie, EMAs were completed on 46.3% of all possible days), the average length of participation was 5 out of a total of 6 possible weeks and suggests that youth received over half of all possible intervention messages. Since intervention messages were only delivered after EMAs were completed, the average participant received messages on less than half of the study days. However, even with this low EMA completion rate and corresponding lower than expected intervention dose (less than 50%), the results indicate that the intervention demonstrated a significant impact on several key outcomes. It is important to consider that while EMA completion rates are much higher for youth EMA studies in general (78%, range 54.6%-96.2%) [52], in an EMA study with YEH, the completion rate was 62% [43]. This is the first study using EMA-responsive JITAI with YEH and, therefore, comparative data are lacking.

In a group-based study with YEH, there was a positive intervention effect on alcohol use, motivation, and condom self-efficacy with less than the prescribed intervention dose (48% completion) [46]. This suggests that a lower than planned dose may still lead to change. While comprehensive, high-dose HIV prevention interventions are effective, they also require high participant burden [105], are expensive to support, and are rarely disseminated widely. Further, while there is little evidence of the effectiveness of all interventions for YEH [106], cognitive-behavioral interventions show marginally positive results [86]. Given the difficulty in engaging YEH in HIV prevention interventions in current health care and social service delivery models, mobile, tailored JITAIs may enhance dissemination in hard-to-reach populations such as YEH. Further, since HIV risk behaviors rarely take place during engagements in care and tend to happen within one’s day, JITAIs allow for interventions to be delivered more proximally at times of heightened risk. Given the intervention effect found with a lower than planned level of intervention engagement, there is a need to determine the minimal effective intervention dose to reduce participant burden.

### Limitations

There are several limitations and challenges to consider when interpreting the findings of this study. First, EMA is an emerging method, and, therefore, there is no robust body of science on EMA measurement psychometrics. However, while no validated EMA scale exists for urge, a systematic review of 91 studies using EMA found the vast majority used a 1-item measure [107]. Further psychometric studies are needed on EMA measures. Second, this was a small pilot study with a convenience sample that only allowed for a preliminary examination of the intervention effects without the power to detect small effect sizes. Third, the control condition received generic motivational messages, which may have positively impacted stress management strategies, thereby diluting any effects that may be attributable to the intervention. Fourth, there may have also been reactivity to EMAs [52] similar to a Hawthorne Effect—HIV health risk behaviors went down over time in both groups, potentially because participants were thinking about their health more due to the EMAs and the generic health messages. Social desirability cannot be eliminated as a possibility that may have affected participant reports. Finally, 16 phones were reported lost or stolen during the study, one was broken, and one was returned after being found in the possession of a different participant. Despite these limitations, the strengths of the study include the diverse gender identity, sexual orientation, and race/ethnicity of the sample.

### Conclusion

In conclusion, the findings from this study suggest a positive effect of a highly scalable mobile intervention that increases access to a HIV prevention intervention for a hard-to-reach population. MY-RID incorporates several of the core elements indicative of apps that have the potential to change behavior, including knowledge and information, goals and planning, feedback and monitoring, and actions [108]. This intervention builds on proven mHealth strategies [76], tailored education [77,78], and motivational messaging [79,80], delivered at the time of heightened need. Prevention interventions tailored for YEH continue to be rare and yet have led to improvements in sexual health outcomes [86,106,109-111]. Therefore, more research is needed to build on YEH’s willingness to participate in intervention research studies [44,45], improvement intervention engagement, and continue to explore the mounting evidence on efficacy. For example, studies are needed to assess whether the survey length and frequency affect engagement rates. However, it is crucial to involve YEH in the development of interventions as studies suggest improved outcomes when programs are tailored and relevant [49].

## Appendix

Multimedia Appendix 1 The MY-RID EMA survey.

Multimedia Appendix 2 Messaging algorithm.

Multimedia Appendix 3 Bayesian stats and sensitivity analysis.

Multimedia Appendix 4 Frequencies and proportions of risky behaviors.

Multimedia Appendix 5 CONSORT-eHEALTH (V 1.6.1) checklist.

## Abbreviations

EMA: ecological momentary assessment
IMB: Information, Motivation, and Behavioral Skills
JITAI: just-in-time adaptive intervention
mHealth: mobile health
MSM: men who have sex with men
MY-RID: Motivating Youth to Reduce Infection and Disconnection
OR: odds ratio
PrEP: pre-exposure prophylaxis
REALM: Rapid Estimate of Adult Literacy in Medicine-Short Form
YEH: youth experiencing homelessness
