# Supplementary material for: Efficacy of a Just-in-Time Adaptive Intervention to Promote HIV Risk Reduction Behaviors Among Young Adults Experiencing Homelessness: Pilot Randomized Controlled Trial
Source: J Med Internet Res. 2021 Jul 6;23(7):e26704. doi: 10.2196/26704 (PMC8292946; doi:10.2196/26704)
Supplement: Multimedia Appendix 1 [file jmir_v23i7e26704_app1.docx]

| **#** | **Survey Item** | **Response Options** |
| --- | --- | --- |
| 1 | Right now, I feel upset. | Strongly disagree (1)  Disagree (2)  Neutral (3)  Agree (4) Strongly agree (5) |
| 2 | Right now, I feel angry. |  |
| 3 | Right now, I feel guilty. |  |
| 4 | Right now, I feel scared. |  |
| 5 | Right now, I feel irritable. |  |
| 6 | Right now, I feel ashamed. |  |
| 7 | Right now, I feel restless. |  |
| 8 | Right now, I feel nervous. |  |
| 9 | Right now, I feel afraid. |  |
| 10 | Right now, I feel stressed. |  |
| 11 | Right now, I feel depressed. |  |
| 12 | Right now, I feel sad. |  |
| 13 | Right now, I feel bored. |  |
| 14 | I am feeling a strong urge to: (check all that apply) | Have sex (1) Do drugs (2) Drink alcohol (3) Steal (4) None of the above (5) |
| 15 | Where did you sleep last night? | Relative or family home(1) Home of friend or acquaintance (2) Home of boyfriend/girlfriend/sexual partner (3) Shelter (4) Street, outside, or abandoned building (5) Bus, metro, train, or car (6) Hotel, motel (7) |
| 16 | Yesterday, how often did you feel that you were unable to control the important things in your life? | Never (1) Almost never (2) Sometimes (3) Fairly often (4) Very often (5) |
| 17 | Yesterday, how often did you feel confident about your ability to handle your personal problems? |  |
| 18 | Yesterday, how often did you feel that things were going your way? |  |
| 19 | Yesterday, how often did you feel that difficulties were piling up so high that you could not overcome them? |  |
| 20 | How many hours of sleep did you get last night? | 0 (1) 1 to 3 hours (2) 4 to 6 hours (3) 7 or more hours (4) |
| 21 | I believe someone discriminated against me today. | Yes (1) No (0) |
| 22 | What was the main reason (s) for the discrimination that you experienced yesterday? (check all that apply) | Your age (1) Your gender (2) Your race, ethnicity, or nationality (3) Your religion (4) A physical disability or other aspect of your appearance (5) Your sexual orientation (6) Being poor or homeless (7) Other (8) |
| 23 | Who discriminated against you yesterday? Check all that apply | Family member (1) Boyfriend (2) Girlfriend (3) Stranger (4) Acquaintance (5) Friend (6) Employer (7) None of the above (8) |
| 24 | Yesterday, I was (check all that apply): | I did not get assaulted yesterday (1) Raped/sexually assaulted (2) Hit, slapped, punched, or kicked (3) Robbed (4) Verbally abused (5) Held against my will (6) |
| 25 | Who assaulted you yesterday? Check all that apply | Family member (1) Boyfriend (2) Girlfriend (3) Stranger (4) Acquaintance (5) Friend (6) Employer (7) |
| 26 | Which of the following behaviors did you do yesterday? Check all that apply | I had sex (1)  I used drugs (2)  I used alcohol (3)  None of the above (4) |
| 27 | What type of sex did you have yesterday? Check all that apply | I did not have sex yesterday (1) Oral (2) Vaginal (3) Anal (4) Other (5) |
| 28 | How many times did you have sex yesterday? | 1 time (1) 2 times (2) 3 times (3)  4 or more times (4) |
| 29 | How many people did you have sex with yesterday? | 1 (1) 2 (2)  3 (3) 4 or more (4) |
| 30 | Who did you have sex with yesterday? Check all that apply | A new sex partner (1) Previous sex partner (2) Boyfriend or girlfriend (3) Stranger (4) Friend or acquaintance (5) Sex worker (6) IV drug user (7) Person who is HIV + (8) Other (9) |
| 31 | What gender was your partner(s) yesterday? Check all that apply | Male (1) Female (2) Non-binary gender/intersex (3) |
| 32 | Did you plan to have sex each time? | Yes (1)  No (0) |
| 33 | Yesterday, did you use a condom each time you had sex? | No, none of the time (1) Yes, some of the time (2) Yes, all of the time (3) |
| 34 | Yesterday, did you or your partner use birth control each time you had sex? |  |
| 35 | What kind of birth control? Check all that apply | Pill (1) Patch (2) Ring (3) Implants (4) IUD (5) Depo shots (6) Condom (7) Other (8) |
| 36 | Yesterday, I traded sex. | Yes (1)  No (0) |
| 37 | What did you trade sex for? Check all that apply | A place to stay (1) Money or gifts (2) Drugs or alcohol (3) Food (4) Clothes (5) Other (6) |
| 38 | Overall, how stressed were you yesterday? | Not at all stressed (1) Somewhat stressed (2) Fairly stressed (3)  Very stressed (4) Extremely stressed (5) |
| 39 | What were you stressed about yesterday? Check all that apply | Money or job (1) Being pregnant or parenting (2) Health of family member/friend (3) Not having a place to stay (4) My health or safety (5) Being hungry (6) Boyfriend/girlfriend issues (7) Drugs or alcohol (8) Other (9) |
| 40 | Select all nicotine products you used yesterday. Check all that apply | I did not use nicotine products yesterday (1) Cigarettes (2) Chewing tobacco or snuff (3) E-cigarettes/vaping (4) Hookah (5) Nicotine patch (6) Nicotine gum or lozenges (7) Cigars/cigarillos (8) |
| 41 | How many cigarettes did you smoke yesterday? | 0= I am not a smoker (1) 0 = I am a smoker but didn’t smoke yesterday (2) 1-5 (3) 6-10 (4) 11-15 (5) 16-20 (6) more than 20 (7) |
| 42 | Select all drugs that you used yesterday. (check all that apply) | I injected drugs (1) Kush/K2 (synthetic marijuana, incense packs) (2) Marijuana (3) Ecstasy (MDMA, Molly) (4) Xanax, valium, pain pills (5) Heroin (6) Cocaine or crack (7) LSD, PCP (8) Other drugs (9) |
| 43 | Did you use: | New/clean needle (1) Used/dirty needle (2) I did not use any needles (3) |
| 44 | Yesterday, how many drinks did you have? (one drink = ONE 12 ounce beer, or ONE 12 ounce wine cooler, or ONE 5 ounce glass of wine, or ONE 1.5 ounce shot, or ONE mixed drink containing 1.5 ounces of liquor) | 0 (1) 1 (2) 2 (3) 3 (4) 4 (5) 5 (6) More than 5 (7) |
| 45 | I worked yesterday. | Yes (1)  No (0) |
| 46 | I attended school yesterday. | Yes (1)  No (0) |
| 47 | I feel completely committed to using a condom every time I have sex. | Strongly disagree (1) Disagree (2) Neutral (3) Agree (4) Strongly agree (5) |
| 48 | I feel completely committed to using birth control every time I have sex to avoid getting pregnant. |  |
| 49 | I feel completely committed to being drug and alcohol free. |  |
| 50 | I find it difficult to stay focused on what's happening in the present and not get distracted. |  |
| 51 | How many servings of fruits and vegetables did you eat yesterday? | 0 (1) 1 (2) 2 (3) 3 (4) 4 (5) 5 or more (6) |
| 52 | How much exercise did you get yesterday? | 0 (1) About 30 minutes (2) About 60 minutes (3) More than 60 minutes (4) |
| 53 | I looked at pornography today. | Yes (1)  No (0) |
| 54 | Are you taking PrEP for HIV prevention? | Yes (1)  No (0) |
| 55 | Did you take your PrEP pill yesterday? | Yes (1)  No (0) |
| 56 | Did you get tested for HIV yesterday? | Yes (1)  No (0) |
| 57 | Were you high or drunk when you had sex? | Yes (1)  No (0) |
