# Supplementary material for: Efficacy of a Just-in-Time Adaptive Intervention to Promote HIV Risk Reduction Behaviors Among Young Adults Experiencing Homelessness: Pilot Randomized Controlled Trial
Source: J Med Internet Res. 2021 Jul 6;23(7):e26704. doi: 10.2196/26704 (PMC8292946; doi:10.2196/26704)
Supplement: Multimedia Appendix 2 [file jmir_v23i7e26704_app2.docx]

Messaging Algorithm

Additional information about the decision process for messaging can be found here. Probabilities for engagement in sex and substance use were calculated using models that we had previously developed (1,2).

**Sexual Behavior Risk**

Generalized linear mixed models from our previous study revealed several predictors of engagement in sexual activity that could increase HIV risk (1). These included constant characteristics (race, sexual orientation), diagnosed conditions (psychosis, PTSD), and time-varying predictors (urge for sex, drug use). The app was designed to automatically read the values of the *k* predictors $x^{(k)}$ and calculate the real-time probability of engagement in the behavior that increased HIV risk:

$$P(sex) = \text{logit}^{-1}\left( \sum\beta^{(k)} x^{(k)} \right).$$

The values of coefficients $\beta^{(k)}$ were known from the aforementioned study and the participant was considered to be at risk when P(sex) > 0.2, based on considerations of the balance of true positives and false positives. The likelihood ratio was estimated to be 3.8 for this decision threshold.

If a participant was classified as being at risk, the app selected a message bin that was tailored to warn about the risks of sexual activity. A message from that bin was selected at random and pushed out to the participant. The availability of multiple messages reduced the chances of repetitive messages that might be ignored. If the participant was classified as being at low risk, a message was pushed out from another bin that was not tailored toward the risks of sexual activity.

**Drug Use Risk**

The estimation of risk and the decision process was analogous to the method described for sexual behavior risk. Predictors of drug use were all time-varying: urge for drug use, urge for alcohol use, urge to steal, viewing pornography, alcohol use, and experience of discrimination (2). The participant was considered to be at risk when the probability of drug use exceeded 0.33, based on considerations of the balance of true positives and false positives. The likelihood ratio was estimated to be 4.0 for this decision threshold.

If a participant was classified as being at risk for drug use, the app selected a message bin that was tailored to warn about the risks of using drugs. A message from that bin was selected at random and pushed out to the participant. The availability of multiple messages reduced the chances of repetitive messages that might be ignored. If the participant was classified as being at low risk for drug use, a message was pushed out from another bin that was not tailored toward the risks of drug use.

**References**

1. Santa Maria D, Padhye N, Yang Y, Gallardo K, Businelle M. Predicting Sexual Behaviors Among Homeless Young Adults: Ecological Momentary Assessment Study. JMIR Public Health Surveill. 2018 Apr 10;4(2):e39.

2. Santa Maria D, Padhye N, Yang Y, Gallardo K, Santos G-M, Jung J, et al. Drug use patterns and predictors among homeless youth: Results of an ecological momentary assessment. Am J Drug Alcohol Abuse. 2018;44(5):551–60.
