# Supplementary material for: Efficacy of a Just-in-Time Adaptive Intervention to Promote HIV Risk Reduction Behaviors Among Young Adults Experiencing Homelessness: Pilot Randomized Controlled Trial
Source: J Med Internet Res. 2021 Jul 6;23(7):e26704. doi: 10.2196/26704 (PMC8292946; doi:10.2196/26704)
Supplement: Multimedia Appendix 3 [file jmir_v23i7e26704_app3.docx]

Appendix 3. Statistical Analysis

Additional information about the longitudinal models, sensitivity analysis, and post-hoc power analysis is provided in this appendix.

**Longitudinal Models**

Bayesian hierarchical logistic regression models (1) were used to assess the intervention effects on engaging in sex, drug use, alcohol use, and their corresponding urges. The time was measured in terms of the day number since starting the study and it was transformed on a log scale (base 2) since data visualization indicated that linearity could be assumed only after transformation. The fixed effects included the main effects of time and the intervention group on the overall odds of the outcome as well as the effect of the intervention through interaction with the time. Random intercept and random slope allowed the log-odds to vary among participants. It is important to note that all EMA data were collected while the intervention was ongoing in one group and participants in the control group were receiving messages designed for the control arm. Therefore, finding a statistically significant main effect of the intervention group can also indicate the presence of an intervention effect, which is not the case in study designs with clearly defined pre- and post- intervention phases. Nevertheless, it is true that there is more confidence in the intervention effect when the interaction of time and intervention group is significant.

Given a binary outcome $y_{ij}\in\left\{ 0,1 \right\}$ for participant *i* and time *j*, the sampling design is specified by the Bernoulli distribution conditional upon the predictors:

$$\text{bernoulli}\left\{ y_{ij} | \text{logit}^{-1}\left( \sum_{k} \beta_{i}^{(k)}.x_{ij}^{(k)} \right) \right\},$$

where there are *k* coefficients $\beta_{i}^{(k)}$ for each subject and $x_{ij}^{(k)}$ are *k* predictors. In particular, the models for intervention effects included a constant term, time, group, and the interaction of time and group. Vague normal priors were used for fixed effects:

$$\beta^{\left( k \right)} \sim\text{normal}(0, 100),$$

while the random intercept and slope coefficients $\beta_{i}^{(k)}$ were sampled from normal prior distributions:

$$\beta^{\left( k \right)}\sim\text{normal}\left( \mu^{\left( k \right)},\sigma^{\left( k \right)} \right).$$

The hyperparameters for the mean were also given vague normal priors:

$$\mu^{\left( k \right)} \sim\text{normal}(0, 100),$$

and the distribution of the scale hyperparameter $\sigma^{\left( k \right)}$ was uniform and restricted to positive values. The Hamiltonian Monte Carlo simulation was implemented in the *RStan* package (2) via code written and executed in the *RStudio* environment (3). Four chains were used for each model with 4000 to 5000 iterations per chain and these were run in parallel on four cores of an Intel i5 processor. The number of iterations were increased, if necessary, until the R̂ measure showed convergence and proper mixing of chains.

Stress experienced at the time of response and stress experienced by participants on the previous day were analyzed similarly after binary recoding of each stress based on its median value. The response to both questions about stress were rated on a 1-to-5 scale. Alternative models that treated the scales as continuous and as ordinal scales with cut points on a latent continuous scale were explored but they did not perform well.

**Sensitivity Analysis**

Sensitivity analysis was conducted to check the robustness of statistically significant intervention effects that resulted from the hierarchical regression models. The models were expected to provide robust intervention effects if data were missing at random. We considered the possibility of two nonignorable missing mechanisms and used a tipping-point approach for the sensitivity analysis (4).

One missingness mechanism that we considered was that participants who never reported a particular risky behavior during the study period may have had a uniform probability of underreporting it over the duration of their participation. That is, participants without a documented habit of risky behavior may have had a uniform probability of occasional indulgence. Another missingness mechanism that we proposed was that participants who reported engaging in a risky behavior during the study period may have a higher probability of that behavior on non-response days than estimated under the missing-at-random assumption.

The model estimates of probability for each participant on each non-response day were modified according to the two missingness mechanisms and data were imputed on the basis of the new probabilities. The tipping-point approach allowed the exploration of the impact of these missingness mechanisms on the intervention effect in a two-dimensional space. One dimension represented the translations of probability estimates, corresponding roughly to an average of 0, 1, 2, 3, and 4 unreported events that were uniformly likely over the study period. The other dimension represented multiplicative factors of 1, 2, 3, and 4 for inflating the probability estimates on non-response days for participants with a record of engaging in the risky behavior. The intervention effect for each cell in the 2-d space was estimated by summarizing 200 imputed results using Rubin’s rules and implemented with the miWQS package for R (5).

**Post-hoc Power Analysis**

The study was originally planned as a feasibility study that would test initial efficacy of the intervention. The sample size was not based on power considerations because there was no precedent for anticipating the effect sizes. At the conclusion of the study, we estimated the post-hoc power for the primary outcomes (having sex, drug use, alcohol use) based on the sampled data. Monte Carlo simulations were carried out using the SIMR package (6) for the longitudinal generalized linear mixed model design. The coefficients for fixed effects in each model along with estimates of the variances of the random effects can be found in Table 3 of the manuscript. The power for the intervention effect on drug use was estimated to be 78% (95% CI: 64% to 88%). The power for intervention effects on sex and alcohol use were 22% (95% CI: 12% to 36%) and 10% (95% CI: 3% to 22%), respectively. The sample sizes for the calculation were N_1_ = 48 in the intervention group and N_2_ = 49 in the control group, while α was set equal to 0.05.

**References**

1. Gelman A, Carlin JB, Stern H, Dunson DB, Vehtari A, Rubin DB. Bayesian data analysis. 2014.

2. Stan Development Team. RStan: the R interface to Stan [Internet]. 2020. Available from: http://mc-stan.org/

3. RStudio Team. RStudio: Integrated Development for R. [Internet]. Boston, MA: RStudio, Inc.; 2019. Available from: http://www.rstudio.com/

4. Liublinska V, Rubin DB. Sensitivity analysis for a partially missing binary outcome in a two-arm randomized clinical trial. Stat Med. 2014 Oct 30;33(24):4170–85.

5. Hargarten PM, Wheeler DC. miWQS: Multiple Imputation Using Weighted Quantile Sum Regression. [Internet]. 2019. (R package). Available from: https://CRAN.R-project.org/package=miWQS

6. Green P, MacLeod CJ. SIMR: an R package for power analysis of generalized linear mixed models by simulation. Methods Ecol Evol. 2016 Apr 1;7(4):493–8.
