# Supplementary material for: Efficacy of a Just-in-Time Adaptive Intervention to Promote HIV Risk Reduction Behaviors Among Young Adults Experiencing Homelessness: Pilot Randomized Controlled Trial
Source: J Med Internet Res. 2021 Jul 6;23(7):e26704. doi: 10.2196/26704 (PMC8292946; doi:10.2196/26704)
Supplement: Multimedia Appendix 4 [file jmir_v23i7e26704_app4.docx]

**Appendix 4**

**Frequencies of Risky Behaviors**

|  | | | **Week** | | | | | |
| --- | --- | --- | --- | --- | --- | --- | --- | --- |
|  | **Group** |  | **1** | **2** | **3** | **4** | **5** | **6** |
| Responses ^a^ | Control | Sum | 256 | 187 | 151 | 138 | 123 | 102 |
|  | Intervention | Sum | 258 | 184 | 159 | 139 | 99 | 80 |
| Drug use ^b^ | Control | Sum | 67 | 30 | 32 | 40 | 29 | 17 |
|  | Intervention | Sum | 70 | 23 | 11 | 7 | 0 | 0 |
| Sex ^b^ | Control | Sum | 59 | 29 | 29 | 23 | 10 | 13 |
|  | Intervention | Sum | 51 | 26 | 13 | 18 | 8 | 4 |
| Alcohol use ^b^ | Control | Sum | 32 | 12 | 13 | 9 | 8 | 7 |
|  | Intervention | Sum | 35 | 11 | 15 | 12 | 4 | 1 |

^a^ Number of responses by treatment group and week

^b^ Number of events reported by treatment group and week

**Proportions of Risky Behaviors**

|  | **Group** |  |  |  |  |  |  |  |
| --- | --- | --- | --- | --- | --- | --- | --- | --- |
| Drug use % ^a^ | Control | Mean | 25.9% | 15.9% | 21.3% | 29.1% | 23.7% | 15.2% |
|  | Intervention | Mean | 26.5% | 12.5% | 7.1% | 4.9% | 0.0% | 0.0% |
| Sex % ^a^ | Control | Mean | 23.0% | 15.5% | 19.2% | 16.9% | 7.6% | 12.8% |
|  | Intervention | Mean | 18.3% | 14.3% | 8.1% | 12.9% | 8.1% | 4.8% |
| Alcohol % ^a^ | Control | Mean | 12.1% | 6.2% | 8.5% | 6.5% | 6.8% | 6.4% |
|  | Intervention | Mean | 12.9% | 5.8% | 9.3% | 8.3% | 4.4% | 1.4% |

^a^ Weekly mean of daily proportion of events reported by treatment group and week
